# Supplementary material for: Integrated Transcriptomic, Metabolomic, and Physiological Analyses Reveal New Insights into Fragrance Formation in the Heartwood of Phoebe hui
Source: Int J Mol Sci. 2022 Nov 14;23(22):14044. doi: 10.3390/ijms232214044 (PMC9698381; doi:10.3390/ijms232214044)
Supplement: Supplementary file 1 [file ijms-23-14044-s001.zip › supplementary figures.pdf]

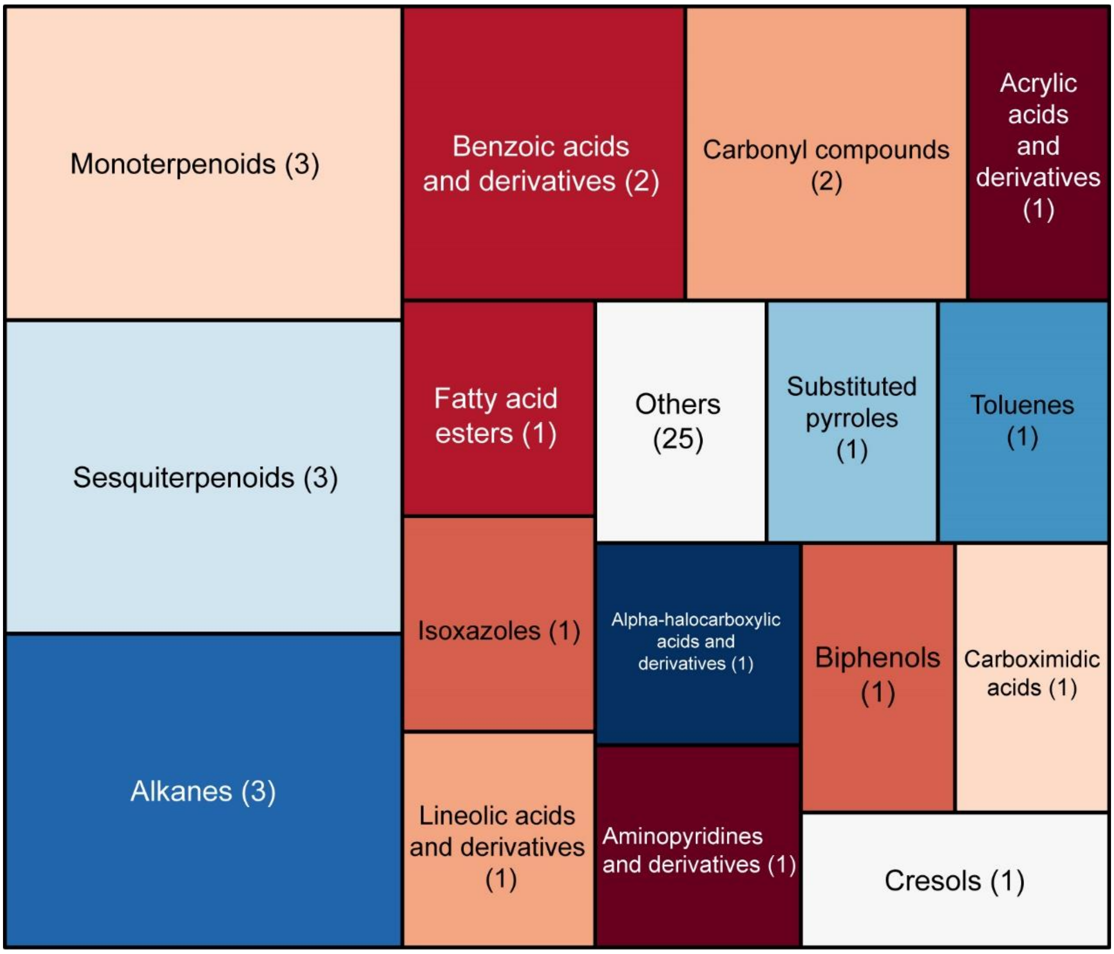

Figure S1. Tree map of metabolite classification

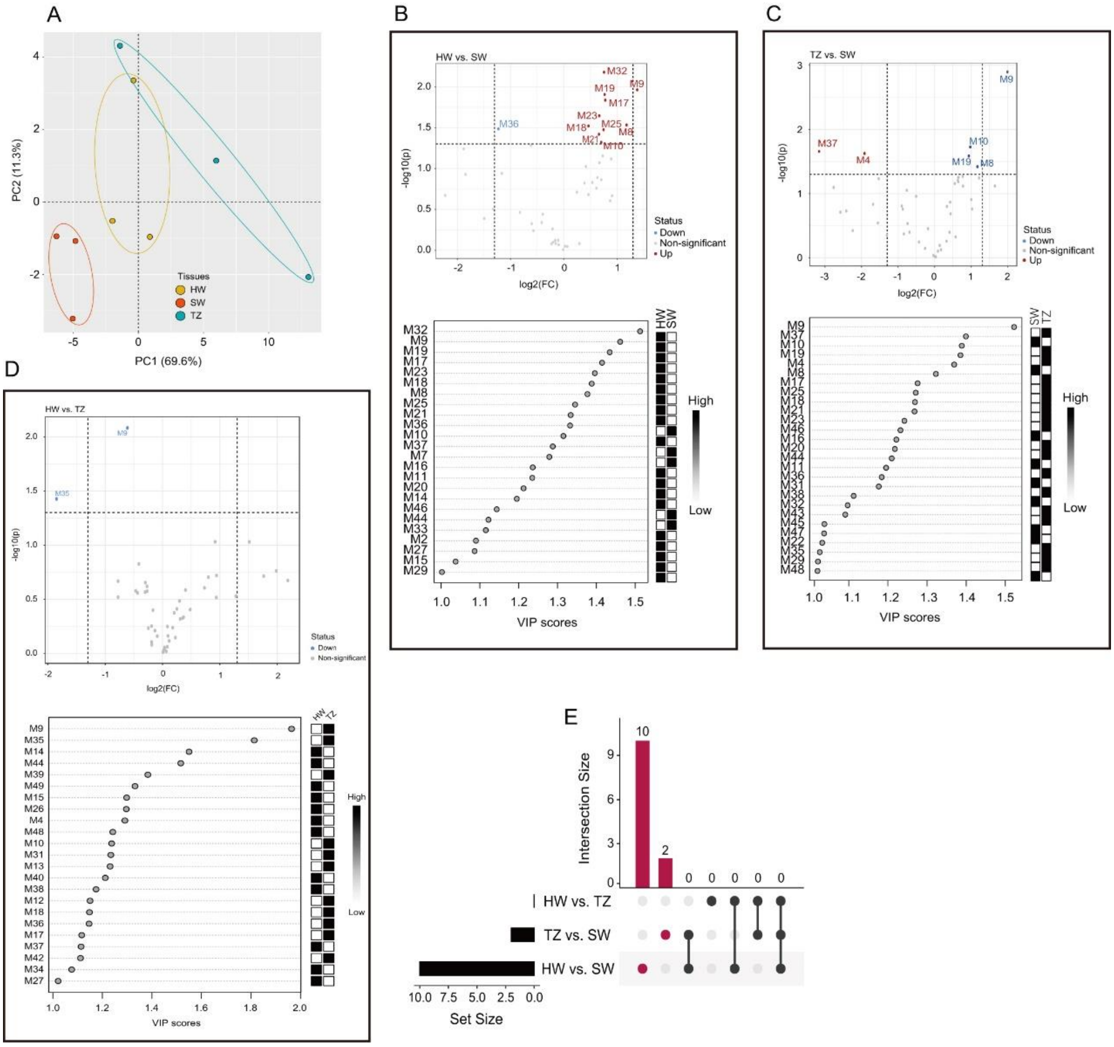

Figure S2. Volatile metabolites and DAMs in *P. hui* heartwood. (**A**) PCA analysis of metabolites. (**B**) Volcano and VIP scores plots of DAMs between HW and SW. The VIP scores showed all DAMs with the VIP ≥ 1. (**C**) Volcano and VIP scores plots of DAMs between TZ and SW. The VIP scores showed all DAMs with the VIP ≥ 1. (**D**) Volcano and VIP scores plots of DAMs between HW and TZ. The VIP scores showed all DAMs with the VIP ≥ 1. (**E**) Venn plot (upset diagram) of DAMs from all comparisons. The red spot and column represented the DAMs only in HW vs. SW or TZ vs. SW

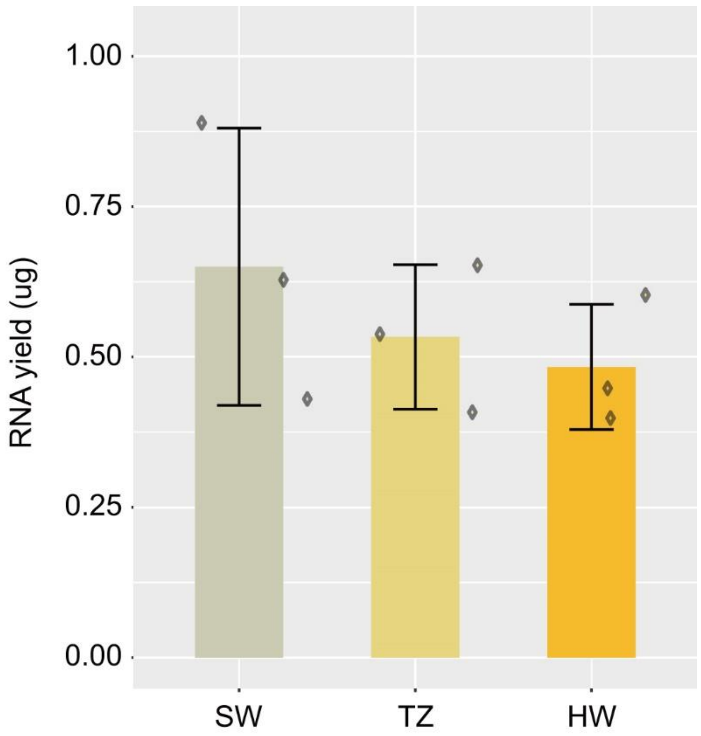

Figure S3. RNA yield of three tissues

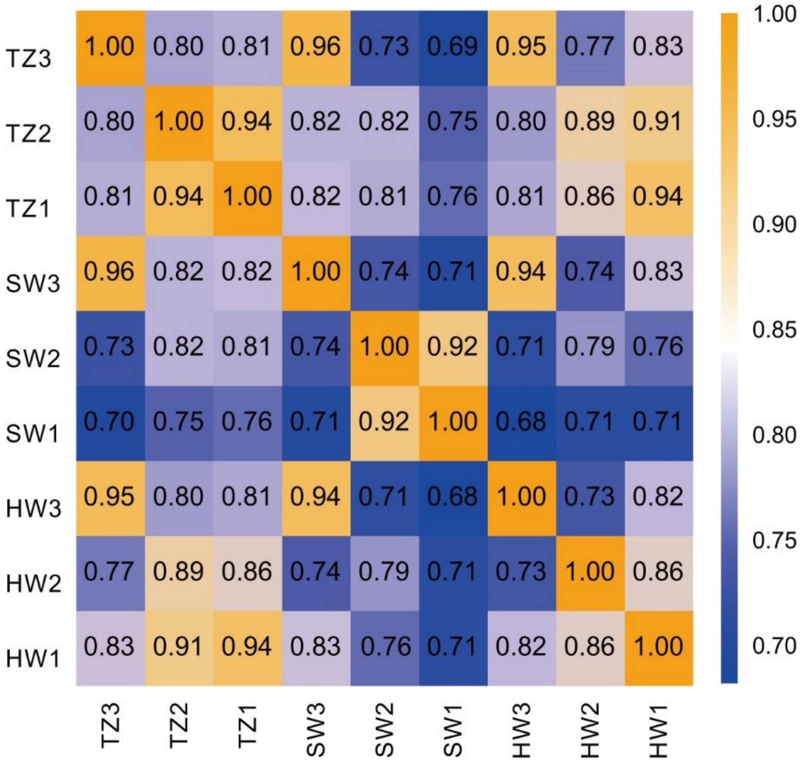

Figure S4. Correlation of transcriptomic data between replicates

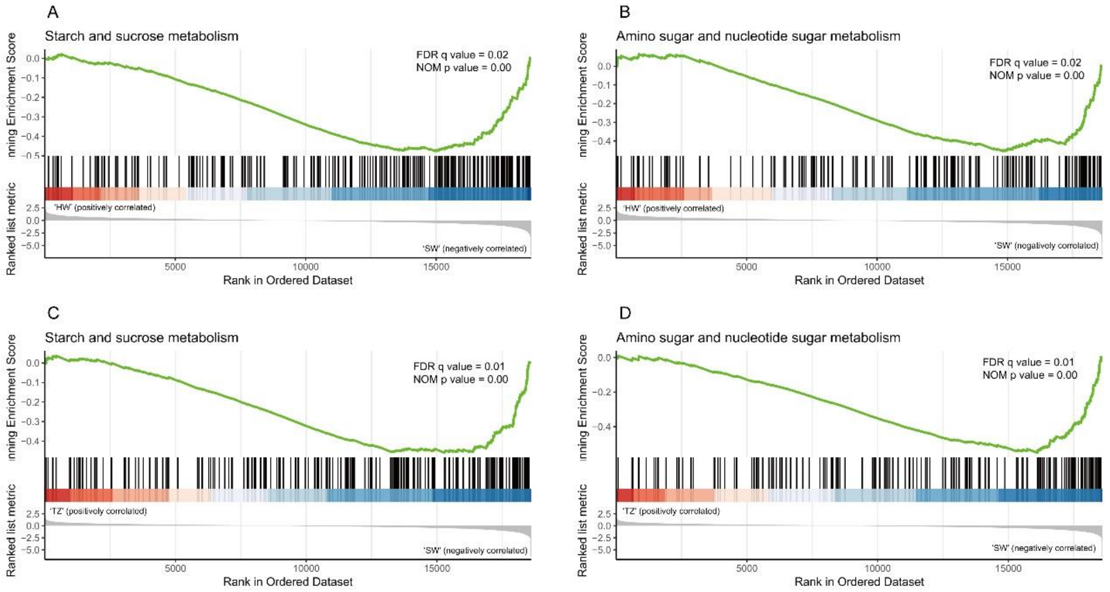

Figure S5. GSEA (gene set enrichment analysis) of starch and sucrose metabolism and amino sugar and nucleotide sugar metabolism genes. (**A**) Starch and sucrose metabolism GSEA rank was calculated by HW vs. SW. (**B**) Amino sugar and nucleotide sugar metabolism GSEA rank was calculated by HW vs. SW. (**C**) Starch and sucrose metabolism GSEA rank was calculated by TZ vs. SW. (**D**) Amino sugar and nucleotide sugar metabolism GSEA rank was calculated by TZ vs. SW

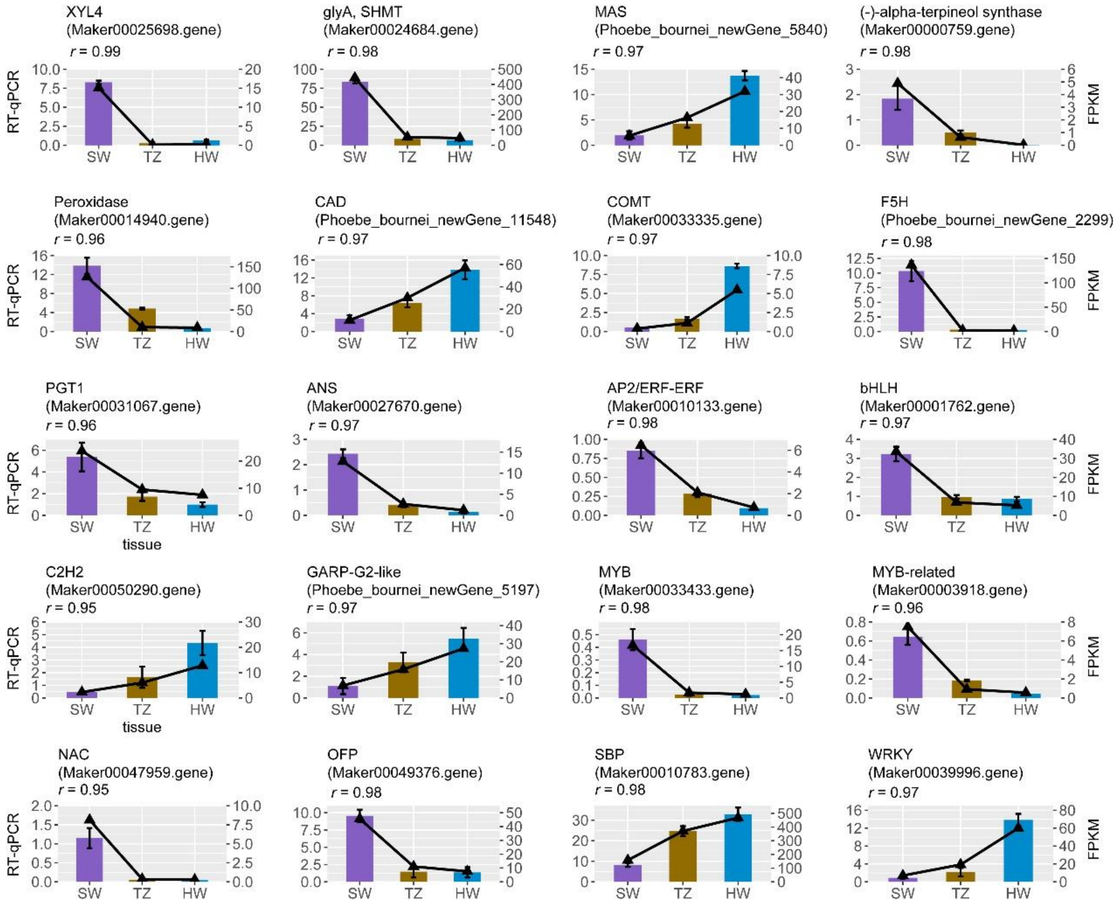

Figure S6. The RT-qPCR analysis of the expression level and fragment per kilobase per million reads (FPKM) of 20 pathway genes and transcription factors genes in HW, TZ, and SW. The column showed the relative expression level, the line showed the FPKM value.
